# Short peptide analogs as alternatives to collagen in pro-regenerative corneal implants

**DOI:** 10.1016/j.actbio.2018.01.011

**Published:** 2018-03-15

**Authors:** Jaganmohan R. Jangamreddy, Michel K.C. Haagdorens, M. Mirazul Islam, Philip Lewis, Ayan Samanta, Per Fagerholm, Aneta Liszka, Monika K. Ljunggren, Oleksiy Buznyk, Emilio I. Alarcon, Nadia Zakaria, Keith M. Meek, May Griffith

**Affiliations:** a Dept. of Clinical and Experimental Medicine, Linköping University, S-58185 Linköping, Sweden; b Dept. of Ophthalmology, Antwerp University Hospital, Wilrijkstraat 10, B-2650 Antwerp, Belgium; c Faculty of Medicine and Health Sciences, Department of Ophthalmology, Visual Optics and Visual Rehabilitation, University of Antwerp, Campus Drie Eiken, Universiteitsplein 1, 2610 Antwerp, Belgium; d Structural Biophysics Group, School of Optometry and Vision Sciences, Cardiff University, Wales CF24 4HQ, UK; e Division of Cardiac Surgery, University of Ottawa Heart Institute, 40 Ruskin Street, Ottawa, ON K1Y 4W7, Canada; f Maisonneuve-Rosemont Hospital Research Centre and Dept. of Ophthalmology, University of Montreal, Montreal, QC H1T 4B3, Canada; g Tej Kohli Cornea Institute, LV Prasad Eye Institute, Hyderabad - 500 034, India

**Keywords:** Collagen-like peptide, Recombinant human collagen, Cornea, Regeneration, Exosomes

## Abstract

Short collagen-like peptides (CLPs) are being proposed as alternatives to full-length collagen for use in tissue engineering, on their own as soft hydrogels, or conjugated to synthetic polymer for mechanical strength. However, despite intended clinical use, little is known about their safety and efficacy, mechanism of action or degree of similarity to the full-length counterparts they mimic. Here, we show the functional equivalence of a CLP conjugated to polyethylene glycol (CLP-PEG) to full-length recombinant human collagen *in vitro* and in promoting stable regeneration of corneal tissue and nerves in a pre-clinical mini-pig model. We also show that these peptide analogs exerted their pro-regeneration effects through stimulating extracellular vesicle production by host cells. Our results support future use of CLP-PEG implants for corneal regeneration, suggesting the feasibility of these or similar peptide analogs in clinical application in the eye and other tissues.

**Statement of significance:**

Although biomaterials comprising full-length recombinant human collagen and extracted animal collagen have been evaluated and used clinically, these macromolecules provide only a limited number of functional groups amenable to chemical modification or crosslinking and are demanding to process. Synthetic, customizable analogs that are functionally equivalent, and can be readily scaled-up are therefore very desirable for pre-clinical to clinical translation. Here, we demonstrate, using cornea regeneration as our test bed, that collagen-like-peptides conjugated to multifunctional polyethylene glycol (CLP-PEG) when grafted into mini-pigs as corneal implants were functionally equivalent to recombinant human collagen-based implants that were successfully tested in patients. We also show for the first time that these materials affected regeneration through stimulation of extracellular vesicle production by endogenous host cells that have migrated into the CLP-PEG scaffolds.

## Graphical abstract

**Figure:**
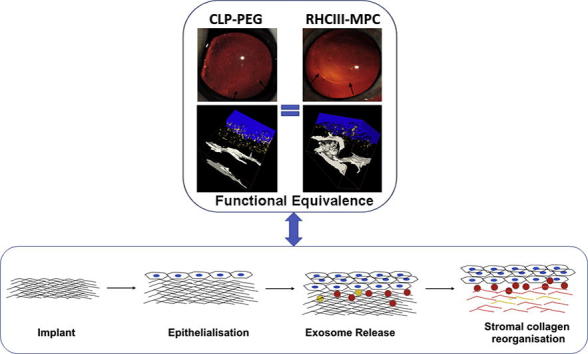

## 1 Introduction

The current global need for replacement organs and tissues requires numbers that far exceed the donor supply. Artificial organs as alternatives can potentially save and improve the quality of lives of patients. The macromolecules of the extracellular matrix (ECM), specifically collagen, have been extensively tested for use as organ substitutes [1]. The premise is that the ECM macromolecules will stimulate regeneration, recapitulating their role in organogenesis during embryogenesis. ECM-derived or inspired biopolymers ranging from decellularized tissues/organs to fabricated constructs are being evaluated or used clinically for promoting regeneration. However, natural ECM macromolecules like collagen are large and difficult to extract and process or modify, and are heterogeneous and source-dependent. While the use of recombinantly produced human collagen eliminates heterogeneity, these are still large macromolecules that require significant processing. This makes short peptide analogs that are readily prepared, easily customized and scaled-up, very attractive ECM alternatives for regenerative medicine. Several *in vitro* and *in vivo* studies have reported that such short ECM-mimicking peptides can stimulate regeneration in a range of organ systems including bone and spinal cord [2], [3]. However, despite their great potential, little is known about their safety and efficacy, mechanism of action, or functional equivalence to their clinically evaluated full-length counterparts.

Here, we tested under pre-clinical conditions in mini-pigs, the safety and efficacy of a collagen-like peptide (CLP) conjugated to an inert but mechanically tougher multifunctional polyethylene glycol (PEG) to emulate the function of a collagen-based implant [4] for promoting regeneration, using the cornea as a model system. The cornea is the transparent front of the eye and major refractive surface for focusing light to the retina for vision. Its superficial location and transparency allow easy access and real-time visualization of cell-biomaterial interactions. A diseased or damaged cornea with permanent transparency loss results in blindness. The conventional treatment is transplantation with donated human corneas. However, like other organs, cornea transplantation suffers from a severe global shortfall, with an estimated 12.7 million patients worldwide awaiting transplantation, or only one out of every 70 patients being transplanted [5]. The transparency, accessibility plus the pressing medical need make the cornea an attractive model tissue.

We previously used full-length recombinant human collagen type III (RHCIII) to fabricate implants that successfully stimulated stable regeneration of the human cornea, an organ that does not normally regenerate on its own. Corneal implants comprising RHCIII only and RHCIII incorporating 2-methacryloyloxyethyl phosphorylcholine (MPC), a synthetic lipid-polymer that can suppress inflammation, [6] were evaluated clinically [7], [8], [9]. They respectively promoted stable corneal regeneration without continual immunosuppression in 10 conventional transplantation patients [7], [8] and three patients diagnosed as being at high-risk for rejection of donor corneas [9]. Although the use of RHCIII instead of extracted animal collagen mitigates potential source heterogeneity, xenogeneic reaction, or risk of pathogen transmission [10], [11], RHCIII replicates full-length collagen, which, is large and relatively difficult to chemically tailor or process, unlike short peptides. In this study, we compared in detail the safety, efficacy, and mechanism of promoting regeneration of corneal implants made from CLP-PEG to control implants made from clinically tested RHCIII-MPC *in vitro*, and in rabbit and mini-pig animal models. Successful testing in a simple organ system like the cornea will allow for the extension to more complex applications such as skin and heart, as we have shown with collagen after minimal modification [12], [13], [14].

## 2 Materials and methods

### 2.1 Hydrogel implants

CLP-PEG and RHCIII-MPC implants were prepared as previously described [4], [15]. Briefly, CLP comprising Cys-Gly-(Pro-Lys-Gly)_4_(Pro-Hyp-Gly)_4_(Asp-Hyp-Gly)_4,_ was synthesized (UAB Ferentis, Vilnius, Lithuania) and conjugated to a 40 kDa 8 arm PEG-maleimide (Creative PEG Works, NC, USA) by continually stirring in ddH_2_O at pH 4.5 for 2 days at a molar ratio of 32:1 and then dialyzed against distilled water using 12–14 kDa MWCO tubing for 2–3 days before lyophilization. 500 mg of 12% (w/w) CLP-PEG solution prepared was crosslinked with EDC and NHS at molar equivalents of CLP-PEG-NH_2_:EDC (1:2) and at equal molar ratio of EDC:NHS. All the reagents were moulded into flat sheets or cornea-shaped implants [4].

RHCIII-MPC implants were fabricated by mixing 18% (w/w) aqueous solution RHCIII (FibroGen Inc., San Francisco, CA) with 2-methacryloyloxyethyl phosphorylcholine (MPC, Paramount Fine Chemicals Co. Ltd., Dalian, China) and poly(ethylene glycol) diacrylate (PEGDA, Mn 575, Sigma-Aldrich) in a morpholinoethane sulfonic acid monohydrate (MES, Sigma-Aldrich, MO) buffer. The ratio of RHCIII:MPC was 2:1 (w/w) and PEGDA: MPC was 1:3 (w/w). Polymerization initiators ammonium persulphate (APS; Sigma-Aldrich) and N,N,N,N-tetramethylethylenediamine (TEMED, Sigma-Aldrich) at ratios of APS:MPC = 0.03:1 (wt/wt), APS:TEMED (wt/wt) = 1:0.77, crosslinker, N-(3-dimethylaminopropyl)-N'-ethylcarbodiimide (EDC; Sigma-Aldrich) and its co-reactant, N-hydroxysuccinimide (NHS; Sigma-Aldrich) was then mixed in. The resulting solution was dispensed into cornea-shaped moulds and cured [15].

After demoulding, all hydrogels were washed thoroughly with phosphate buffered saline (PBS) and placed into vials of aseptic PBS containing 1% chloroform, which were sealed to maintain sterility.

### 2.2 Physical and mechanical characterization

Flexure tests, physical appearance and light transmittance measurements were carried out following the established quality control (QC) protocols [15], [16]. The same tests were used to determine stability of the implant after storage for a minimum of 12 months at 4 °C. Qualitative analysis included Fourier Transform Infrared (FTIR) spectroscopy to evaluate the chemical integrity of the components within the hydrogel implants after 12 months of storage. The most relevant amide IR vibrations for CLP were used for this purpose: Amide A, B, I and II [17]. Samples were dried for 3 days. Measurements were carried out in a Nicolet 6700 FTIR spectrometer equipped with a Smart iTR Attenuated Total Reflectance (ATR) sampling accessory with 4 cm^−1^ resolution; a total of 64 individual spectra were collected for each sample. Representative spectra were collected in a Nicolet iS5 FT-IR spectrometer equipped with an iD7 ATR accessory using 300 individual spectra with 4 cm^−1^ resolution.

Collagenase from *Clostridium histolyticum* (Sigma-Aldrich, MO, USA), at 5 U/ml in 0.1 M Tris-HCl buffer containing 5 mM CaCl_2_ was used to evaluate the stability of hydrogels as previously described [4]. Briefly, samples were weighed after blotting off surface water at different time points to determine the rate of loss of mass. The percentage of residual weight was calculated using the following equation: Residual mass % = W_t_/W_o_%, where W_t_ is the weight of hydrogel at a certain time point and W_o_ is the initial weight of the hydrogel.

Denaturation temperature of the CLP-PEG hydrogels was determined using a Q2000 differential scanning calorimeter (TA Instruments, New Castle, DE), with a temperature range from 8 to 200 °C at a scan rate of 5 °C min^−1^. Hydrogels (approximately 3 to –5 mg) were placed in an aluminum pan after removing the surface water. The pan was then hermetically sealed to make an airtight condition. Denaturing temperature was calculated from the curve of heat flow versus temperature increase.

The refractive indices of CLP-PEG hydrogels were measured at 19 °C at a wavelength of 589 nm, using a bench-top Abbe 60 series Refractometer (Bellingham and Stanley, London, UK) that was calibrated against a silica test plate of known refractive index.

For rheology, three, six-month old female New Zealand White rabbits, were implanted with CLP-PEG hydrogels, 6 mm in diameter, 350 µm thick by anterior lamellar keratoplasty after ethical permission from local ethical committee, Linköpings Djurförsöksetiska Nämnd and in compliance with Swedish Animal Welfare Ordinance and Animal Welfare act. At 6 months post-operation, the rabbits were euthanized. Operated corneas and their unoperated contralateral corneas were dissected. Oscillatory rheology was performed on a discovery hybrid rheometer, DHR-2 from TA instruments (Sweden). Circular disks of 8 mm diameter were cut out from the excised rabbit eyes (operated and unoperated) using a biopsy punch. All experiments were performed using 8 mm diameter parallel-plate geometry and at 35 °C under axial force of 170mN to ensure firm gripping. All frequency sweep experiments were performed using shear strain amplitude of 0.27%.

### 2.3 In vitro toxicology and biocompatibility

#### 2.3.1 Vitotox™ASSAY

Observations on cytotoxicity and genotoxicity were done with the previously described Vitotox™ protocol (Gentaur, Belgium) [18]. In brief, Vitotox™ is closely related to the Ames test and is regarded as a highly sensitive technique for cytotoxic and genotoxic screening. A diluted TA104 RecN2-4 (Genox) and TA104 (Cytox) *Salmonella typhimurium* suspension (1/10 dilution) was added to homogenized RHCIII-MPC and CLP-PEG. S9 was added to the designated +S9 cultures to test the genotoxic effects of the metabolites of the compounds. The bacterial suspensions were then incubated, shaking at 36 °C, and the luminescent signal was measured every 5 mins for 4 h. Signal to Noise ratio (S/N value) were recorded for Genox and Cytox respectively. Scaffolds were soaked in 0.1 M PBS for 24 h and homogenized in a tissue grinder for 5 min and sonicated for 40 min. Three concentrations of the resulting homogenate were tested (100% (undiluted), 10% (1/10) and 1% (1/100) v/v). A reduction in the S/N value (<0.8 cut-off) of the luminescent signal of –S9 suspensions indicates cytotoxicity, whereas a genotoxic effect is characterized by an increase (>1.5 cut-off) of the luminescent signal of +S9 suspensions. 4-nitroquinoline-oxide (4-NQO) and benzo[α]pyrene (BaP) are used as positive controls for Genox strain. BaP only turns genotoxic after S9 metabolization (+S9).

#### 2.3.2 Metabolic activity and live/dead cell viability assay

RHCIII-MPC and CLP-PEG hydrogels of 6 mm diameter were placed into 96-well plates. Immortalized human corneal epithelial cells (HCECs) [19] were seeded onto the surface of the materials at a density of 9000 cells per hydrogel (n = 3). Cells seeded at similar density on tissue culture plastic (TCP) served as a control. HCECs were cultivated in keratinocyte serum-free medium (KSFM; 17005075, Thermo Fisher Scientific, MA, USA) in a humidified 37 °C incubator for 4 days (96 h). At 24 h, 48 h, 72 h and 96 h of cultivation, a PrestoBlue cell metabolic activity assay (A13261, Thermo Fisher Scientific) was performed according to the manufacturer’s protocol. In brief, PrestoBlue was added to the cultures and incubated for 60 min. The supernatant was transferred to a 96 well plate and fluorescence was read at 590 nm using VICTOR3 plate reader (Perkin Elmer, MA, USA). Finally, cultures were rinsed 1× with preheated KSFM to remove excess PrestoBlue, after which the cultures were further cultivated in KSFM. Supplementary Live/Dead staining (L3224, Thermo-Fisher Scientific) was performed at 48 h cultivation, where cells were double-stained with calceinacetoxymethyl (Calcein AM) and ethidium homodimer-1 (EthD-1). Cells cultured on TCP and treated with 0.1% Saponin for 30 min at 20 °C were used as positive controls for the Live/Dead assay. Independent non-parametric testing was performed using the SPSS 21 Kruskal-Wallis test (IBM Corp, NY, USA) and Prism 5 (GraphPad software, CA, USA). P ≤ .05 was considered significant.

### 2.4 Corneal implant surgery and clinical evaluation in mini-pigs

In preparation for clinical translation, in compliance with the OECD Principle of Good Laboratory Practice (GLP), ENV/MC/CHEM (98) 17, 1997, and with local ethical permission, one CLP-PEG or RHCIII-MPC implant was grafted into the right cornea of each of four Gottingen mini-pigs (Ellegaard, Denmark) per group by anterior lamellar keratoplasty (ALK), at Adlego Biomedical AB (Solna, Sweden). Two weeks before surgery the animals were given a thorough clinical examination to establish a baseline for corneal health. Animals were intubated and anaesthetized prior to surgery. The right cornea of each pig cornea was cut with a 6.5 mm circular trephine to a depth of 500 µm, and the corneal button was then manually dissected with a diamond knife and removed. Hydrogel implants were cut with 6.75 mm diameter trephine and placed into the wound bed. A piece of 7 mm diameter clinical human amniotic membrane (HAM) (St:Erik’s Eye Hospital, Stockholm) was placed over the implant to suppress undesired inflammation and the implants were kept in place with 10/0 nylon overlying sutures. Upon completion of the surgery, an antibacterial and anti-inflammatory ophthalmic suspension (Tobraone with 3 mg/ml dexamethasone and 1 mg/ml tobramycine, Alcon, Sweden) was administered. The maintenance dose was 1 drop, 3 times daily for 5 weeks. The unoperated contralateral corneas served as controls.

The health status of all animals was monitored throughout the 12 months study. The corneas and implants were evaluated before surgery, at 5 weeks and then at 3, 6, 9 and 12 months after surgery. The examinations were performed by a surgeon who was blinded to the specifications of the implants in individual animal. These examinations consisted of slit lamp biomicroscopy (to evaluate haze from a 0 to +4 scale, any neovascularization and general health of the eye using modified MacDonald-Shadduck scoring system [20], pachymetry to measure cornea/implant thickness (Tomey SP 3000, Tomey, Inc., Japan), Schirmer’s tear test to evaluate the extent of the tear film (tear strips from TearFlo, Hub Pharmaceuticals, Rancho Cucamonga, CA, USA), intraocular pressure (using a TonoVet tonometer, Icare Finland Oy, Vantaa, Finland), esthesiometry to determine corneal sensitivity as a measure of nerve function (using a Cochet-Bonnet esthesiometer; Handaya Co., Tokyo, Japan) and *in vivo* confocal microscopy (Heidelberg HRT3 with a Rostock Cornea Module, Heidelberg Engineering GmbH, Dossenheim, Germany) to access in-growth of corneal cells, nerves and any blood vessels or inflammatory cells.

### 2.5 Immunohistochemistry and immunocytochemistry

Samples of implanted and control unoperated corneas were processed through a sucrose gradient for frozen sectioning. Seven micron frozen sections mounted on glass slides were fixed with cold methanol (10 min, −20 °C), air dried, immersed in PBS and then incubated with 5% goat serum (Cat. No. 0060-01, Southern Biotech, Birmingham, AL) in Tris buffered saline (TBS) with 0.1% saponin (incubation buffer) over night at 4 °C. Subsequently, sections were incubated with a range of the primary antibodies diluted with incubation buffer overnight at 4 °C. The antibodies tested are listed in Table S1.

After washing off residual primary antibodies with incubation buffer, sections were incubated with the secondary antibodies (goat anti-rabbit Alexa 488 or goat anti-mouse Alexa 488 or goat anti-rabbit Alexa 594; Jackson Immuno Research Laboratories Inc., West Grove, PA) diluted 1:1000 with the blocking solution for 60 min at room temperature. After washing in TBS-T (TBS with 0.05% tween), the slides were dehydrated and mounted with Vectashield mounting medium with DAPI (Vector Laboratories, Inc., Burlingame, CA) for cell nuclei visualization. Fluorescent images were captured using an inverted LSM-800 Zeiss confocal microscope (LSM800, Carl Zeiss Microscopy, Germany). Similar staining procedure was performed for *in vitro* experiments using primary HCEC out grown from the human corneal rim explants on the respective hydrogels or TCP after fixation with 4% paraformaldehyde for 10 min and ice cold Methanol for 10 min at −20 °C. This study using cadaveric human corneal rim explants followed the tenets of the Declaration of Helsinki and was approved by the Antwerp University Hospital Ethical Committee (ethical committee approval: 14/30/319).

To examine the extent of nerve regeneration within the implants, whole mount staining was performed using anti-beta III tubulin antibody. Freshly excised corneas were washed in 0.01 M PBS and fixed in 2% glutaraldehyde and 2% PFA in 0.1 M phosphate buffer (PB) for 3 h, then washed three times in 0.01 M PBS and stored at 4 °C. Before the overnight incubation at 4 °C with the beta III tubulin antibody all specimens were rocked for 20 min in 0.1 M Tris-buffered saline (TBS), mounted in 1% Paraformaldehyde solution for 30 min then washed with TBS, permeabilized with 0.2% Triton X100 (Cat. No. X100, Sigma-Aldrich) in TBS and then blocked for 4 h at room temperature in 4% heat-inactivated fetal bovine serum (FBS) and 0.2% TritonX100 in 0.1 M TBS (blocking solution). Incubation with the secondary goat anti-mouse Alexa 488 antibody diluted 1:1000 with the blocking solution was carried out for 4 h at 4 °C. Finally, specimens were washed with 0.1 M TBS and mounted with DAPI. Pictures were taken using an inverted confocal microscope.

### 2.6 Transmission and scanning electron microscopy

The samples were processed for regular and ultra-low temperature freezing TEM and SBF-SEM. For SBF-SEM, after fixation, samples of implanted corneas were cut into thin (<1 mm) slices to preserve the positioning of the implant in relation to host cornea. Each sample slice was transferred to 1.5% potassium ferricyanide/1% osmium tetroxide in cacodylate buffer for 1 h and then washed in distilled water. The samples were then placed sequentially in 1% aqueous thiocarbohydrazide, 1% osmium tetroxide and 1% aqueous uranyl acetate, each for 1 h. All the staining steps were followed by 30 min distilled water washing steps. The samples were then incubated for 1 h in a solution of lead aspartate at 60 °C. After staining, the corneal samples were washed in two changes of distilled water for 30 mins. The specimens were then dehydrated in an ethanol series from 70% through to 100% and via propylene oxide infiltrated and embedded in CY212 (TAAB, Berkshire, UK) epoxy resin and polymerized for 24 h at 60 °C. The surface of polymerized resin blocks were then trimmed and attached to Gatan (PEP6590) specimen pins. The pins were then gold coated and transferred to a Zeiss Sigma VP FEG SEM equipped with a Gatan 3View2 system, where data sets of up to 1000 images were acquired of the block surface every 100 nm through automated sectioning. Each image was acquired at 4 K X 4 K pixels, at a pixel resolution 6.5 nm and a pixel dwell time of 8 µs. Using an SEM accelerating voltage of 3.4 keV in low vacuum variable pressure mode (28 Pa). Imaging data was acquired from a 26.5 µm × 26.5 µm region of interest. Selected serial image sequences were extracted from the image data and 3D reconstructions were generated using Amira 6.0 software (FEI, ThermoFisher).

For conventional TEM, ∼110 nm ultrathin sections were taken from the central region of the implant specimens prepared for SBF SEM using a Leica UC6 ultramicrotome. The ultrathin sections were collected onto uncoated 300HEX grids.

For optimal structural preservation, RHCIII-MPC and CLP-PEG biosynthetic constructs were processed for TEM using a high pressure freezing technique using a Leica EMPACT2 high pressure freezer (Leica Microsystems GmbH, Vienna, Austria). Approximately 1.5 mm × 1.5 mm × 150 µm of the RHCIII-MPC samples were dissected out to fit into a Leica high-pressure freezer membrane carrier. Vitrified specimens were then freeze substituted at −80 °C in 2% osmium/acetone, 1% tannic acid/acetone and 1% uranyl acetate/acetone for 88 h using a Leica AFS2 freeze substitution device. The samples were then embedded in Araldite resin at 60 °C for 24 h. Ultrathin sections (∼110 nm) were cut and then placed on 1 mm peloform-coated slot grids.

For the visualization of sulphated PGs, the corneas which had been pre-fixed in 2.5% glutaraldehyde/2% paraformaldehyde in 100 mM cacodylate buffer pH 7.2 were washed in 25 mM sodium acetate buffer (pH 5.7) with 0.1 M magnesium chloride for 12 h on a rotator. The corneal samples were then incubated in 0.05% cupromeronic blue in 25 mM sodium acetate buffer (pH 5.7) with 0.1 M magnesium chloride for 12 h at 4 °C. The corneal samples were then briefly washed in buffer followed by 15 min incubations in aqueous 0.5% sodium tungstate, then 0.5% sodium tungstate in 50% ethanol, to enhance the electron density of the PG-cupromeronic blue complex. Specimens were dehydrated through an ascending ethanol series before being embedded in Araldite resin and polymerized at 60 °C for 24 h. Ultrathin sections (∼110 nm thick) were cut with a diamond knife on a Leica Ultracut UC6 ultramicrotome. Sections were collected on uncoated 300HEX copper grids, stained with saturated aqueous uranyl acetate for 30 min at room temperature. All specimens were examined in a JEOL 1010 TEM (Jeol (UK) Ltd.) at an accelerating voltage of 80KV fitted with an 11-megapixel 14-bit Orius SC 1000 CCD TEM camera (Gatan, Pleasanton, CA).

### 2.7 Statistical analyses

Data in table and graphs are reported as means ± SD. Statistical analyses performed for each test is noted in the relevant section. Statistical significance was set at P ≤ 0.05.

## 3 Results

### 3.1 CLP-PEG and RHCIII-MPC hydrogels

**Fig. 1:**
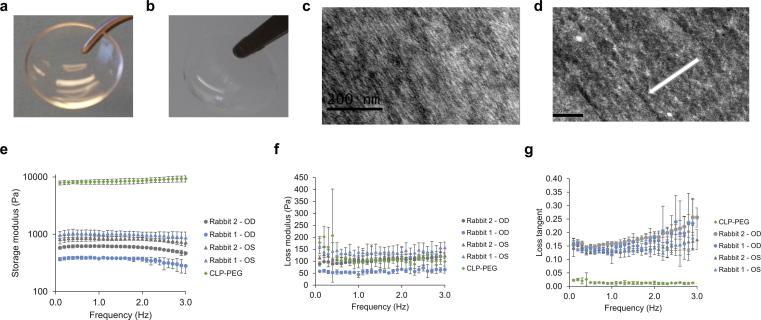
Implants, structure and mechanical stability. Control RHCIII-MPC implants (a) and CLP-PEG implants (b) are both optically clear as 500 μm thick corneal shaped implants. Similarly, mimicking RHCIII-MPC implants (c) CLP-PEG implants (d) display a nanofibrillar structure when observed using high pressure freezing TEM imaging. Scale bars, 200 nm. Oscillatory rheology of CLP-hydrogels pre- and post-grafting into rabbit corneas compared to healthy, unoperated rabbit corneas. (e) Storage modulus, (f) Loss modulus and (g) Loss tangent as a function of oscillation frequency at 0.27% shear strain amplitude.

Both control RHCIII-MPC implants (Fig. 1a) and CLP-PEG implants (Fig. 1b) were optically clear, with over 90% light transmission, exceeding that of the human cornea [21] and a refractive index that approximates that of the human cornea [22] (Table S2A). High pressure freezing transmission electron microscopy (TEM) showed that both hydrogels comprised very fine fibrils (Fig. 1c, d). The denaturation temperature of the CLP-PEG hydrogels was considerably higher than those of native corneas and RHCIII-MPC controls (Table S2A). However, this likely reflects the temperature at which PEG and not the peptides start to melt. RHCIII-MPC implants had considerably higher tensile strength, but CLP-PEG implants were more elastic and showed 4 times the % elongation, compensating for the lower strength during surgical handling (Table S2B). Both implants were significantly weaker than human corneas, but this can be accounted for by the difference in solids content. Implants comprised of well over 90% water while the human cornea contains about 78% water [23].

The CLP-PEG hydrogels were stable in storage for over 12 months at 4 °C, showing no to minimal differences in optical transparency, water content and flexibility (Table S2). Their collagenase degradation profile (Fig. S1A) was similar to that previously reported for freshly-made samples in Islam et al. [4]. FTIR spectra (Table S3, Fig. S1B) between freshly made samples and stored samples shows the presence of all characteristic amide absorption peaks relevant to CLP and are in good agreement with earlier studies [17], [24]: amides A (≈3300 cm^−1^), B (≈3080 cm^−1^), I (≈1660 cm^−1^) and II (≈1500 cm^−1^).

Importantly, CLP-PEG implants grafted into rabbit corneas became stably integrated by six months post-operation. Oscillatory rheology of CLP-PEG hydrogels showed that the storage modulus values of the implants were much higher than those of the rabbit corneas, indicating higher stiffness and lower compliance of the implants (Fig. 1e and f). On the contrary, healthy unoperated rabbit corneas were compliant and extremely tear resistant, with a higher loss tangent, correlating with the damping behavior of a viscoelastic material (Fig. 1 g). The regenerated neo-corneas had mechanical properties similar to those of the healthy naive rabbit corneas (Fig. 1e–g).

### 3.2 In vitro toxicology and biocompatibility evaluation

**Fig. 2:**
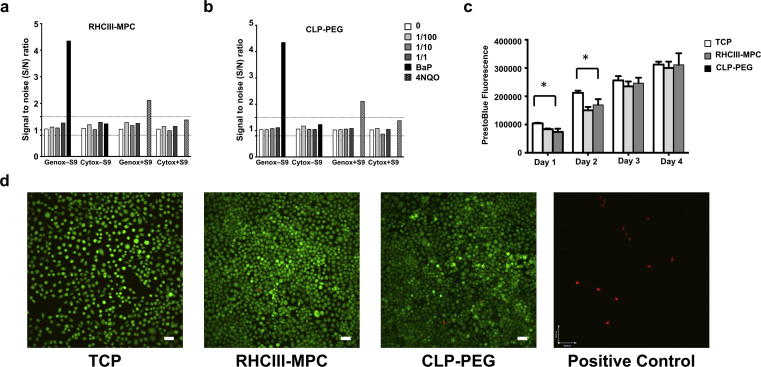
Biocompatibility of Implants. Neither RHCIII-MPC (a) nor CLP-PEG (b) homogenized hydrogels tested at dilutions of 10% (1/10 w/v) and 1% (1/100 w/v) showed any noticeable trace of genotoxicity or cytotoxicity unlike the respective positive controls, BaP and 4NQO. A PrestoBlue assay (c) showed that proliferation rates of human corneal epithelial cells on RHCIII-MPC and CLP-PEG hydrogels were similar. Live/dead assay further shows minimal cell death among the RHCIII-MPC and CLP-PEG like TCP control (d). Scale bars, 100 µm. For a positive staining of Live/dead assay the TCP cultured cells are treated with 0.1% saponin for 30 min at 20 °C.

*In vitro* cytotoxicity and genotoxicity assessments were performed using the Vitotox™ protocol (Gentaur, Belgium), a highly sensitive technique that is closely related to the Ames test. In this test, two different strains of *Salmonella typhimurium* used respectively for cytotoxicity and genotoxicity testing were exposed to different dilutions of homogenized hydrogels, along with positive controls [25]. Neither the RHCIII-MPC controls (Fig. 2a) nor CLP-PEG gels (Fig. 2b) were cytotoxic or genotoxic. PrestoBlue Cell Viability reagent used to assess viability and proliferation showed that there was no statistically significant difference (P > .05) in metabolic activity of human corneal epithelial cells (HCECs) seeded on CLP-PEG or RHCIII-MPC hydrogels (Fig. 2c). The only difference observed was between cells grown on the three-dimensional hydrogels and those grown on two-dimensional tissue culture plastic (TCP) (Fig. 2c). The metabolic activity of cells on TCP was significantly higher at 24 h and 48 h indicating a larger cell number. However, by 72 h and 96 h, there were no differences observed amongst all tested conditions (Fig 2c). These results were confirmed by a supplementary Live/Dead analysis performed at 48 h post-culture, showing minimal cell death on the hydrogels and TCP substrates (Fig. 2d).

### 3.3 Implantation and clinical evaluation

**Fig. 3:**
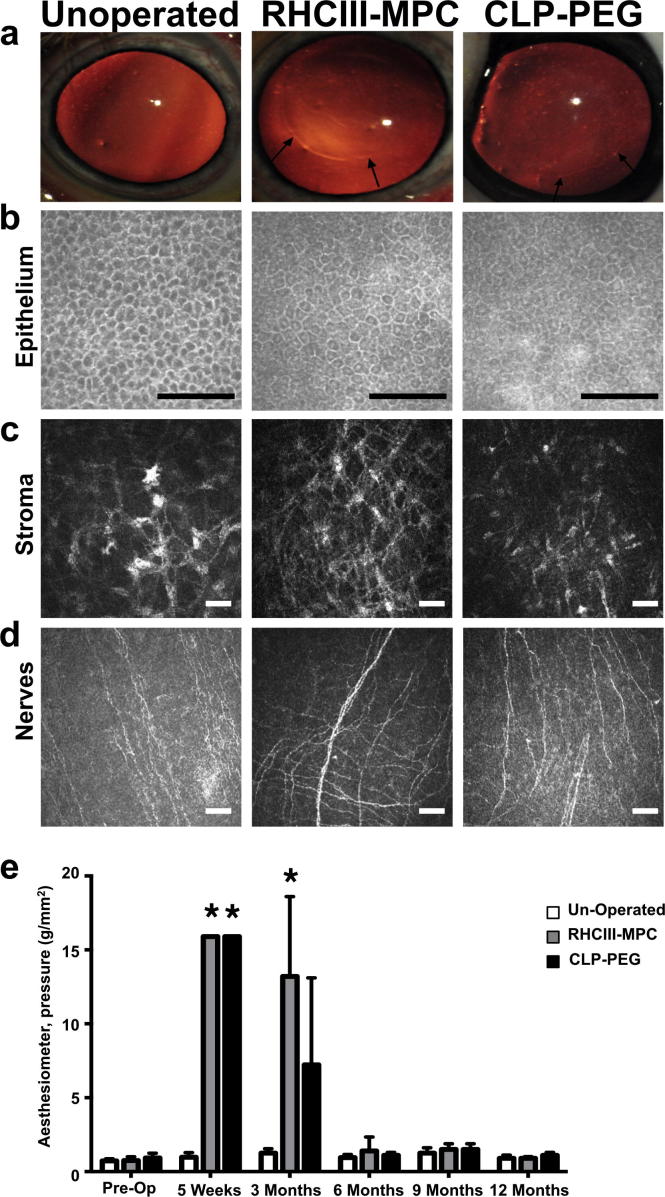
RHCIII-MPC and CLP-PEG corneal implants and their performance in mini-pigs. Examples of optically clear RHCIII-MPC and CLP-PEG hydrogel implants (a). After 12 months of implantation in corneas of mini-pigs, the implants remain clear like the unoperated cornea. Arrows indicate the boundaries of the implants. *In vivo* confocal microscopy shows that both RHCIII-MPC and CLP-PEG implanted corneas have regenerated their epithelium (b), stroma (c) and sub-epithelial nerve plexus (d) to resemble their counterparts in the normal, healthy cornea. Scale bars, 150 µm. Aesthesiometry measures the pressure needed to obtain a blink reaction, i.e. touch sensitivity, which is correlated to nerve function. Pre-operatively, all healthy corneas showed a response to light touch. At 5 weeks post-operation, the implanted corneas were non-responsive, even with maximal pressure exerted. At 3 months, touch sensitivity is returning so less pressure was needed for a response. By 6 months, sensitivity was back to normal levels. ^*^ – p < .05 as compared to un-operated eyes (Kruskal-Wallis test with Bonferroni post hoc test).

Both control RHCIII-MPC and CLP-PEG implants were seamlessly incorporated into the mini-pig corneas without any excessive redness, swelling or inflammation. The rate and course of regeneration stimulated by CLP-PEG implants paralleled that of control RHCIII-MPC implants over the 12-month observation period (Table 1), resulting in neo-corneas of comparable thickness (Fig. S2). At 12 months post-operation, all regenerated neo-corneas remained stably integrated and were optically transparent (Fig. 3a) without any sustained immune suppression. In general, implanted corneas showed early vascular in-growth at three months post-operation, which was coincidental with the development of haze in the grafted eyes. The vessels receded and the implanted corneas became clear again. *In vivo* confocal microscopy showed that all implanted corneas had regenerated epithelia similar to healthy unoperated corneas. Infiltration of stromal cells was observed at three months post-operation (Fig. S3). The corneal nerves that were cut during the surgery had regenerated and were observed penetrating the implant area at six months post-operation (Fig. S3). By 12 months post-operation, the regenerated neo-corneas had fully regenerated epithelia (Fig. 3b), a populated stroma (Fig. 3c) and corneal nerves (Fig. 3d) resembling those of their normal, unoperated contralateral counterparts. Aesthesiometry measures corneal sensory function and hence, provides an indicator of nerve regeneration. At five weeks post-operation, the corneas were unresponsive to touch (Fig. 3e). At three months post-operation, CLP-PEG implanted corneas were more touch sensitive than RHCIII-MPC implanted ones. By six months post-operation, however, touch sensitivity of all implanted corneas was comparable to that of normal controls.

**Table 1:** Progress of CLP-PEG and RHCIII-MPC implants grafted into corneas of mini-pigs, compared to untreated contralateral control eyes over the 12 month post-operation period.

Treatment	Time	Cornea thickness (mm)	Haze	Vascularization		Schirmer's tear test (mm)	Aesthesiometry (mm)	IOP (mmHg)
				implant	margin			
CLP-PEG	pre-op	706 ± 20	0	0	0	12 ± 3	3.9 ± 0.3	10 ± 1
	1 m	–	0.38	0.5	1.75	7 ± 1	0	–
	3 m	690 ± 16	0.25	1.75	0	13 ± 3	1.3 ± 0.4	16 ± 2
	6 m	702 ± 22	0	0	0.75	12 ± 1	3.4 ± 0.1	16 ± 2
	9 m	724 ± 13	0	0	0.5	16 ± 2	2.9 ± 0.1	19 ± 8
	12 m	747 ± 8	0	0	2	13 ± 4	3.4 ± 0.1	22 ± 2

RHCIII-MPC	pre-op	704 ± 21	0	0	0	11 ± 3	4.4 ± 0.6	11 ± 1
	1 m	–	0.38	0.5	0.5	7 ± 2	0	–
	3 m	652 ± 9	0.69	1.75	0.5	15 ± 2	0.4 ± 0.5	14 ± 2
	6 m	669 ± 22	0	0	2	13 ± 2	4.4 ± 0.6	20 ± 2
	9 m	687 ± 11	0	0	0	13 ± 4	4.4 ± 0.7	16 ± 2
	12 m	693 ± 23	0	0	0	15 ± 4	4.4 ± 0.8	15 ± 1

Untreated	pre-op	725 ± 11	0	0	0	8 ± 2	8 ± 2	11 ± 1
	1 m	–	0	0	0	9 ± 1	9 ± 1	–
	3 m	730 ± 10	0	0	0	12 ± 2	12 ± 2	17 ± 2
	6 m	738 ± 10	0	0	0	10 ± 1	10 ± 1	17 ± 3
	9 m	747 ± 11	0	0	0	13 ± 3	13 ± 3	16 ± 6
	12 m	767 ± 11	0	0	0	15 ± 2	15 ± 2	18 ± 2

### 3.4 *Histology and immunohistochemistry*

**Fig. 4:**
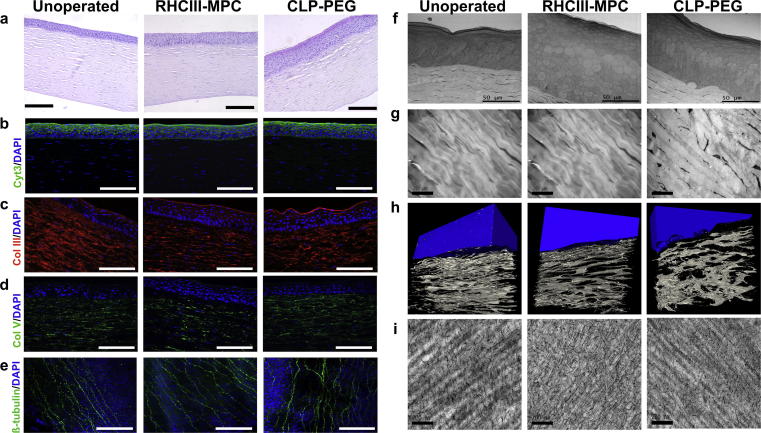
Characterization of regenerated neo-corneas. (a) H&E sections through a healthy, unoperated cornea, and regenerated neo-corneas at 12 months after implantation with control RHCIII-MPC and CLP-PEG scaffolds. Epithelial hyperplasia was noted in the implanted corneas, which is normal in post-grafting tissues (b). Scale bars, 100 µm. The regenerated corneal epithelium shows staining with cytokeratin 3/12, a marker of differentiated cells in all three samples. Stromal collagens types III (c) and V (d) are present in the implanted as well as unoperated corneas, showing in particular that remodeling and extracellular matrix production is occurring in CLP-PEG implants that contained no collagen. Sub-epithelial nerve plexus stained with b-tubulin (e) showing nerve regeneration in CLP-PEG hydrogels as in control RHCIII-MPC and unoperated contralateral eyes. Scale bars, 100 µm. At the ultrastructural level, TEM micrographs of all three samples show an epithelium with distinct layers of elongated, cuboidal and flattened cells, characteristic of healthy corneas (f). Scale bar, 50 µm. TEM images show a regular, lamellar arrangement in both unoperated and regenerated stromas (g). Scale bar, 20 µm. The CLP-PEG neo-corneas have slightly less regular stromas as seen in 3D reconstructed SBF-SEM images (epithelium rendered in blue) (h) suggesting an on-going process. Both neo-corneas contained collagen fibrils decorated with proteoglycans (i), similar to the matrix of the healthy, unoperated cornea. Scale bar, 200 nm.

Histopathological examination of implanted corneas performed in compliance with OECD GLP principles by a blinded vet pathologist (BioVet AB, Sollentuna, Sweden) concluded that there was no difference between CLP-PEG and RHCIII-MPC implanted corneas. Epithelial hyperplasia was noted in all regenerated neo-corneas but otherwise, they resembled unoperated, healthy mini-pig corneas (Fig. 4a).

Both CLP-PEG and RHCIII-MPC regenerated neo-corneas had cytokeratin 3-positive, fully differentiated epithelia, like un-operated controls (Fig. 4b). The regenerated neo-cornea stromas were positively stained for collagen type III (Fig. 4c) which co-exists with type I within the stromal ECM [26], and type V (Fig. 4d), which makes up 10–20% of corneal collagen [27]. No blood vessels (no CD31 staining) or lymphatic vessels (no LYVE1 staining) were found within the regenerated neo-corneas or healthy controls (Fig. S4). β-tubulin staining of whole mounts confirmed restoration of parallel nerve fibres of the sub-epithelial nerve plexus in both implant groups that resembled the unoperated controls (Fig. 4e).

TEM confirmed the similarity of epithelial cells of CLP-PEG and RHCIII-MPC regenerated neo-corneas to those of the unoperated corneas but with hyperplasia (Fig. 4f). The underlying regenerated stromas contained cells arranged in lamellae (Fig. 4 g). However, the lamellae were less structured in the neo-corneas compared to controls, and particularly in the CLP-PEG implanted corneas as seen by serial block face-SEM (SBF-SEM) 3D reconstruction (Fig. 4h; Videos S1–3). Staining for proteoglycans below the newly regenerated epithelium as visualised by TEM showed that their arrangement within RHCIII-MPC and CLP-PEG was similar to the arrangement in an unoperated mini pig cornea (Fig. 4i).

### 3.5 Implant – host interactions

**Fig. 5:**
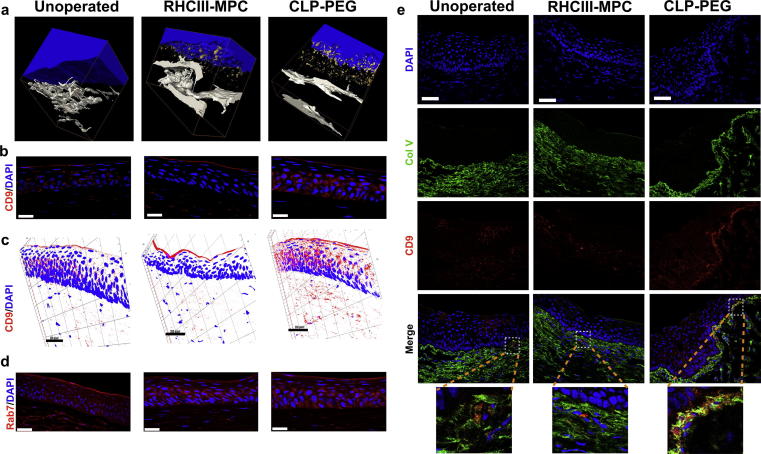
Extracellular vesicle release from regenerating neo-corneas stimulated by biomimetic implants. (a) 3D reconstructed serial block face scanning electron micrographs of an unoperated healthy cornea and regenerating neo-corneas at 12 months after implantation with control RHCIII-MPC or CLP-PEG, showing the release of extracellular vesicles (rendered in gold) from the lower epithelial layers into the stroma. (b) Immunofluorescent micrographs of exosome marker CD-9 stained cornea samples. (c) Corresponding 3D reconstructions of *CD9*-stained sections confirm that the extracellular vesicles released by the epithelium include positively-stained exosomes. (d) *Rab7* stained endosomes-exosomes in both unoperated and implanted corneas. Cell nuclei are counterstained blue with DAPI. Scale bars, 25 µm.

Ultrastructural analysis of CLP-PEG hydrogel-implanted corneas showed that vast quantities of extracellular vesicles (EVs) were secreted from the regenerated corneal epithelium (Fig. 5a, Fig. S5, Video S4). Immunohistochemistry performed on frozen sections of pig corneas implanted with CLP-PEG or RHCIII-MPC showed differential staining for CD9, a well-known marker for exosomes, and Rab-7, a endosome-exosome marker [28]. Healthy unoperated corneas and RHCIII-MPC implanted corneas showed minimal to moderate staining for CD9 while CLP-PEG implanted corneas showed marked CD9 staining (Fig. 5b and c). Rab7 staining was increased in the epithelial layer of CLP-PEG implanted corneas relative to unoperated controls (Fig. 5d). In RHCIII-MPC implants, Rab7 staining was confined to the basal portions of the epithelium. We confirmed that HCECs were producing EVs by culturing these cells on CLP-PEG hydrogels. Controls comprised RHCIII-MPC hydrogels and tissue culture plastic (Fig. S6). HCECs on tissue culture plastic showed CD9 staining mainly at the leading edges. CD9 stained vesicles were seen over the entire cytoplasm of cells grown on RHCIII-MPC, while staining in cells on CLP-PEG were mainly perinuclear (Fig. S5). Rab7 stained vesicles remained intracellular and were markedly increased in HCECs cultured on CLP-PEG, compared to RHCIII-MPC, while staining was perinuclear on tissue culture plastic (Fig. S6). Finally, we observed increased CD9 staining below the basal epithelium of the limbus showing co-localization with collagen type V in the CLP-PEG implanted corneas but neither RHCIII-MPC nor unoperated corneas showed such striking co-localization (Fig. 5e).

## 4 Discussion

A wide range of mimetic peptides have been developed as analogs to native collagen. The Hartgerink group produced the first collagen mimetic peptides that formed hydrogels from purely peptide interactions suggesting their possible functional equivalence to full-length collagen [29]. However, these hydrogels as described were too soft to be used as corneal implants. By conjugating CLP to inert PEG [2], [4], we produced implants that were sufficiently robust for surgical handling [4]. This is keeping with reports where several backbone structures such as poly(amidoamine)-based dendrimers (PAMAM), 4 arm or 8 arm poly ethylene glycol (PEG) when conjugated to CLPs, which not only promoted triple helical formation of the attached CLPs but also provided additional mechanical strength [2]. CLP-PEG hydrogels were optically transparent and flexible, and retained their optical clarity and flexibility after 12 months of storage. Their composition remained unchanged as evidenced by the FTIR spectral profiles of freshly-made and 30-month old hydrogel samples. Although the Amide I absorption peak (primarily peptide bond C = O stretch) is known to be highly dependent on peptide secondary structure [24], [30], the appearance of this very characteristic peak along with the others, namely Amide A, B, and II (Table S3, Fig. S1B), attributed to the presence of CLP which are also in good agreement with previously published reports [17], [24], and the striking similarity in the fingerprint region (≤800 cm^−1^) between the fresh and the stored sample indicate that their chemical composition remained essentially the same. We also showed that the ability of hydrogels to resist collagenase digestion was unchanged after 30 months of storage compared to the profile reported for freshly-made samples [4]. It is pertinent to note, however, that while FTIR and collagenase digestion assays are useful techniques to monitor the stability of the hydrogels, for a complete assessment of long-term storage, complementary methods (such as weight loss, mechanical properties including stress/strain curves, surface changes using SEM, contact angle and analysis of degradation solutions, etc.) would be necessary [31], [32]. Nevertheless, our results indicated that CLP-PEG hydrogels remained sufficiently stable when stored for over 12 months. They were not as tough as the clinically evaluated RHCIII-MPC implants, but compensated for this by being more elastic, which allowed them to withstand the grafting procedure. Implants into rabbit corneas showed that the mechanical properties of the regenerated neo-corneas were similar to healthy, unoperated corneas. This is the desired end-point, so initially weaker implants were not an issue.

Prior to pre-clinical use, a range of *in vitro* toxicology tests is required to demonstrate the safety of the implants. Like RHCIII-MPC implants that have been tested in animals [33] and clinically [7], [9], CLP-PEG implants were cell friendly and allowed for proliferation. Genotoxicity tests results showed little/no risk of induction of mutagenesis.

Unlike RHCIII-MPC implants, however, CLP-PEG implants do not contain any collagen. Remarkably, high pressure freezing TEM showed that CLP-PEG hydrogels had very fine fibrillar structures similar to those of the RHCIII-MPC hydrogels. This supports the contention that CLPs mimic their native counterparts in forming tiny fibrils [34].

When grafted into pig corneas, like the control RHCIII-MPC implants, CLP-PEG implants promoted regeneration of corneal epithelium, stroma and nerves [4]. Here, detailed histopathological examination showed hyperplasia of the epithelium in implanted corneas. The implants were only 500 μm thick while the pigs’ corneas were all over 600 μm, showing that the epithelium had thickened to restore normal corneal thickness. This is a well-known post-surgical finding in human and animal corneas after photorefractive surgery [35], [36] and after corneal implantation [37], so was not considered a pathological finding.

In Islam et al., we reported the presence of type I and V collagen in regenerated neo-corneas implanted with CLP-PEG [4]. In the cornea, as with the rest of the body, the cells most frequently associated with synthesis and secretion of collagen are the fibroblasts and their precursors, although there were early reports that the corneal epithelium secretes collagen [38], [39]. In this study, SBF-SEM clearly showed that the regenerating epithelium produced copious amounts of EVs. Immunohistochemistry showed co-localization of exosome marker CD9 with collagen-type V to these EVs and adjacent area. This strongly suggested that cornea-specific collagen V was secreted by the epithelium into the stroma through EVs. TEM showed that proteoglycans were present. During early cornea development, the epithelium produces ECM that attracts stromal cells. Taken together, our results strongly suggest that this process is recapitulated in the biomaterials-induced corneal regeneration observed, and EV secretion is involved. The regenerated epithelium had most likely secreted collagen and proteoglycans within the implants, thereby attracting in-growth of fibroblasts and nerves. Consistent with this hypothesis is the fact that markedly more exosomes are secreted by epithelium in CLP-PEG implanted corneas that contain no collagen that in control RHCIII-MPC implants where collagen is already present within the scaffolds. While further work is ongoing to characterize the EVs produced by CLP-PEG and RHCIII-MPC implantation, we have nevertheless shown that EVs have a role in collagen- and analog-enabled corneal regeneration. This is in keeping with observations that exosomes are potent mediators of regeneration that are now being tested as cell-free therapeutic agents in several systems including cardiac regeneration [40].

Studies of nerve regeneration showed that corneas implanted with CLP-PEG regained sensitivity slightly faster. However, by six months post-operation, touch sensitivity which is correlated to nerve function was equivalent in both CLP-PEG and control RHCIII-MPC eyes.

## 5 Conclusion

As peptide analogs of ECM proteins head towards clinical application, establishing their safety and efficacy profiles is critical. We have shown that hydrogels made from a short collagen analog conjugated to PEG are safe and functionally equivalent to those comprising full-length, recombinantly produced human collagen that successfully regenerated human corneas, in both *in vitro* and *in vivo* pre-clinical animal testing. We further show that these ECM-mimetic hydrogels promoted regeneration by stimulating in-growing endogenous host cells to produce matrix components through secretory extracellular vesicles. With this safety and efficacy profile and mechanism of action now known, further testing of CLP-PEG hydrogels in human subjects is merited to determine the full extent of the utility of peptide analogs as pro-regeneration implants in the cornea and in other organ systems.

## Appendix A Supplementary data

Supplementary Video S1 Unoperated cornea. Epithelium is pseudocoloured blue.

Supplementary Video S2 RHCIII-MPC implanted cornea.

Supplementary Video S3 CLP-PEG implanted cornea.

Supplementary Video S4 Extracellular vesicles (gold colour) in CLP-PEG implanted cornea.
