# Corrigendum to “Short peptide analogs as alternatives to collagen in pro-regenerative corneal implants” [Acta Biomaterialia 69 (2018) 120–130]

**DOI:** 10.1016/j.actbio.2018.10.009

**Published:** 2018-11

**Authors:** Jaganmohan R. Jangamreddy, Michel K.C. Haagdorens, M. Mirazul Islam, Philip Lewis, Ayan Samanta, Per Fagerholm, Aneta Liszka, Monika K. Ljunggren, Oleksiy Buznyk, Emilio I. Alarcon, Nadia Zakaria, Keith M. Meek, May Griffith

**Affiliations:** a Dept. of Clinical and Experimental Medicine, Linköping University, S-58185 Linköping, Sweden; b Dept. of Ophthalmology, Antwerp University Hospital, Wilrijkstraat 10, B-2650 Antwerp, Belgium; c Faculty of Medicine and Health Sciences, Department of Ophthalmology, Visual Optics and Visual Rehabilitation, University of Antwerp, Campus Drie Eiken, Universiteitsplein 1, 2610 Antwerp, Belgium; d Structural Biophysics Group, School of Optometry and Vision Sciences, Cardiff University, Wales CF24 4HQ, UK; e Division of Cardiac Surgery, University of Ottawa Heart Institute, 40 Ruskin Street, Ottawa, ON K1Y 4W7, Canada; f Maisonneuve-Rosemont Hospital Research Centre and Dept. of Ophthalmology, University of Montreal, Montreal, QC H1T 4B3, Canada; g Tej Kohli Cornea Institute, LV Prasad Eye Institute, Hyderabad 500 034, India

The authors regret that they missed a detail in the Materials and Methods, section, 2.1 Hydrogel implants. Although the CLP sequences were identical, the peptides were synthesized at two different places using the same chemistry. So, to clarify, the first two sentences of the first paragraph should read as follows:

“CLP-PEG and RHCIII-MPC implants were prepared as previously described for the mini-pig studies [4,15]. For all other studies, briefly, CLP comprising Cys-Gly-(Pro-Lys-Gly)4(Pro-Hyp-Gly)4(Asp-Hyp-Gly)4, was synthesized (UAB Ferentis, Vilnius, Lithuania) and conjugated to a 40 kDa 8 arm PEG-maleimide (Creative PEG Works, NC, USA) by continually stirring in ddH2O at pH 4.5 for 2 days at a molar ratio of 32:1 and then dialyzed against distilled water using 12–14 kDa MWCO tubing for 2–3 days before lyophilization.”

There is also a transcription error in Table 1, where data entered for “Aesthesiometry” is incorrect. We have now corrected this error in the revised Table 1 below.

**Table 1:** Progress of CLP-PEG and RHCIII-MPC implants grafted into corneas of mini-pigs, compared to untreated contralateral control eyes over the 12 month post-operation period.

Treatment	Time	Cornea thickness (mm)	Haze	Vascularization		Schirmer's tear test (mm)	Aesthesiometry (mm)	IOP (mmHg)
				Implant	Margin			
CLP-PEG	Pre-op	706 ± 20	0	0	0	12 ± 3	3.9 ± 0.3	10 ± 1
	1 m	–	0.38	0.5	1.75	7 ± 1	0	–
	3 m	690 ± 16	0.25	1.75	0	13 ± 3	1.3 ± 0.4	16 ± 2
	6 m	702 ± 22	0	0	0.75	12 ± 1	3.4 ± 0.1	16 ± 2
	9 m	724 ± 13	0	0	0.5	16 ± 2	2.9 ± 0.1	19 ± 8
	12 m	747 ± 8	0	0	2	13 ± 4	3.4 ± 0.1	22 ± 2

RHCIII-MPC	Pre-op	704 ± 21	0	0	0	11 ± 3	4.4 ± 0.6	11 ± 1
	1 m	–	0.38	0.5	0.5	7 ± 2	0	–
	3 m	652 ± 9	0.69	1.75	0.5	15 ± 2	0.4 ± 0.4	14 ± 2
	6 m	669 ± 22	0	0	2	13 ± 2	3.3 ± 0.4	20 ± 2
	9 m	687 ± 11	0	0	0	13 ± 4	2.9 ± 0.2	16 ± 2
	12 m	693 ± 23	0	0	0	15 ± 4	3.8 ± 0.1	15 ± 1

Untreated	Pre-op	725 ± 11	0	0	0	8 ± 2	4.4 ± 0.3	11 ± 1
	1 m	–	0	0	0	9 ± 1	4.1 ± 0.4	–
	3 m	730 ± 10	0	0	0	12 ± 2	3.4 ± 0.1	17 ± 2
	6 m	738 ± 10	0	0	0	10 ± 1	3.9 ± 0.1	17 ± 3
	9 m	747 ± 11	0	0	0	13 ± 3	3.3 ± 0.3	16 ± 6
	12 m	767 ± 11	0	0	0	15 ± 2	3.6 ± 0.2	18 ± 2

The authors apologize for any confusion or inconvenience caused.

