# Corrigendum to “Short peptide analogs as alternatives to collagen in pro-regenerative corneal implants” [Acta Biomater. 69 (2018) 120–130]

**DOI:** 10.1016/j.actbio.2019.01.046

**Published:** 2019-04-01

**Authors:** Jaganmohan R. Jangamreddy, Michel K.C. Haagdorens, M. Mirazul Islam, Philip Lewis, Ayan Samanta, Per Fagerholm, Aneta Liszka, Monika K. Ljunggren, Oleksiy Buznyk, Emilio I. Alarcon, Nadia Zakaria, Keith M. Meek, May Griffith

**Affiliations:** a Dept. of Clinical and Experimental Medicine, Linköping University, S-58185 Linköping, Sweden; b Dept. of Ophthalmology, Antwerp University Hospital, Wilrijkstraat 10, B-2650 Antwerp, Belgium; c Faculty of Medicine and Health Sciences, Department of Ophthalmology, Visual Optics and Visual Rehabilitation, University of Antwerp, Campus Drie Eiken, Universiteitsplein 1, 2610 Antwerp, Belgium; d Structural Biophysics Group, School of Optometry and Vision Sciences, Cardiff University, Wales CF24 4HQ, UK; e Division of Cardiac Surgery, University of Ottawa Heart Institute, 40 Ruskin Street, Ottawa, ON K1Y 4W7, Canada; f Maisonneuve-Rosemont Hospital Research Centre and Dept. of Ophthalmology, University of Montreal, Montreal, QC H1T 4B3, Canada

The authors regret that an error was published in the original version of Fig. 4. A corrected figure, along with its original caption, is provided below.

**Fig. 4:**
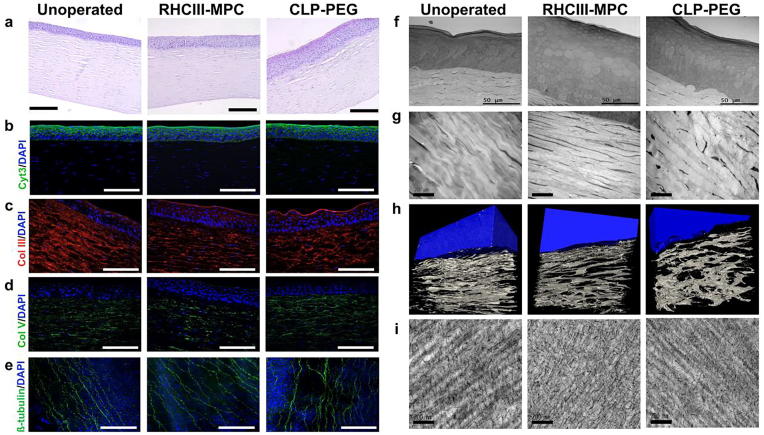
Characterization of regenerated neo-corneas. (a) H&E sections through a healthy, unoperated cornea, and regenerated neo-corneas at 12 months after implantation with control RHCIII-MPC and CLP-PEG scaffolds. Epithelial hyperplasia was noted in the implanted corneas, which is normal in post-grafting tissues (b). Scale bars, 100 µm. The regenerated corneal epithelium shows staining with cytokeratin 3/12, a marker of differentiated cells in all three samples. Stromal collagens types III (c) and V (d) are present in the implanted as well as unoperated corneas, showing in particular that remodeling and extracellular matrix production is occurring in CLP-PEG implants that contained no collagen. Sub-epithelial nerve plexus stained with b-tubulin (e) showing nerve regeneration in CLP-PEG hydrogels as in control RHCIII-MPC and unoperated contralateral eyes. Scale bars, 100 µm. At the ultrastructural level, TEM micrographs of all three samples show an epithelium with distinct layers of elongated, cuboidal and flattened cells, characteristic of healthy corneas (f). Scale bar, 50 µm. TEM images show a regular, lamellar arrangement in both unoperated and regenerated stromas (g). Scale bar, 20 µm. The CLP-PEG neo-corneas have slightly less regular stromas as seen in 3D reconstructed SBF-SEM images (epithelium rendered in blue) (h) suggesting an on-going process. Both neo-corneas contained collagen fibrils decorated with proteoglycans (i), similar to the matrix of the healthy, unoperated cornea. Scale bar, 200 nm. (For interpretation of the references to colour in this figure legend, the reader is referred to the web version of this article.)

The authors apologize for any confusion or inconvenience caused.

