# Supplementary material for: Short peptide analogs as alternatives to collagen in pro-regenerative corneal implants
Source: Acta Biomater. 2018 Mar 15;69:120–30. doi: 10.1016/j.actbio.2018.01.011 (PMC5842042; doi:10.1016/j.actbio.2018.01.011)
Supplement: Supplementary Data [file mmc5.docx]

**Appendix A. Supplementary Data**

**Supplementary Tables**

Table S1. Antibodies used in immunohistochemical analyses.

| **Antibody** | **Dilution** | **Specificity** | | |
| --- | --- | --- | --- | --- |
| Anti-AE-5(K3/K12) antibody  Abcam, Cambridge, UK | 1:50 | Differentiated cornea epithelial cells | | |
| Anti Collagen V antibody  Abcam, Cambridge, UK | 1:500 | | type V collagen found in cornea | |
| Anti- Collagen III antibody  Abcam, Cambridge, UK | 1:100 | | type III collagen | |
| Anti Lyve-1, Abcam (ab33682), Cambridge, UK | 1:100 | | | lymphatic vessels |
| Anti CD31, polyclonal antibody, Abcam (ab28364), Cambridge, UK | 1:50 | | | endothelium of blood vessels |
| Anti-tubulin beta III antibody, Invitrogen (480011), Camarillo, CA, USA | 1:200 | | | neuronal marker |
| Anti-CD9 antibody  BioRad, CA, USA | 1:50 | | | exosome marker |
| Anti-Rab7 antibody  Abcam, Cambridge, UK | 1:100 | | | endosome marker |

**Table S2**: Properties of CLP-PEG hydrogels compared to the RHCIII-MPC hydrogels they are designed to replace as corneal implants.

A. Performance according to QC specifications, and comparison to human corneas.

| Properties | Transmission at 500 nm (%) | Back-  scatter (%) | Refractive index | Denaturation  Temperature  (°C) | Water content (%) | Diameter  (mm) | Flexure (mm) |
| --- | --- | --- | --- | --- | --- | --- | --- |
| RHCIII-MPC Implants | 92.1 ± 0.1[1] | 1.66 ± 0.55[1] | 1.334 ± 0.00[1] | 56.96 ± 1.05[1] | 85.5 ± 0.2[2] | 12 ± 0.0 | 6 ± 0.0 |
| CLP-PEG | 92.4 ± 0.95[3] | 0.90 ± 0.17[3] | 1.34 ± 0.00 | 151.30 ± 9.91 | 91.65 ± 1.10[3] | 12 ± 0.0 | 6 ± 0.0 |
| Human cornea | 87.1 ± 2.0  [4] | <3[5] | 1.373-1.380  [6] | 65.1  [7] | 78[8] | 12 mm | n/a |

B. Mechanical properties of hydrogels and comparison to human corneas.

| Implant | Tensile Strength  (MPa) | Elongation  (%) | Modulus  (MPa) |
| --- | --- | --- | --- |
| RHC-III-MPC | 0.26 ± 0.06[1] | 12.15 ± 0.84[1] | 3.63 ± 0.84[1] |
| CLP-PEG | 0.07 ± 0.02[3] | 58.30 ± 4.49[3] | 0.18 ± 0.06[3] |
| Human cornea | 3.81 ± 0.40[6] | **-** | 3-13[9] |

Table S3. Stability study for CLP-PEG implants stored for 12 months in phosphate saline buffer pH 7.4 at 4-8ºC showed that optical transmission, flexibility, size and appearance did not change.

| Sample ID | Flexure test | Physical appearance | Transmittance 400-700 nm | FTIR* | | | |
| --- | --- | --- | --- | --- | --- | --- | --- |
|  |  |  |  | Amide A | Amide B | Amide I | Amide II |
| 20150629KD110.1 | Pass | Pass | 88.4 | Yes | Yes | Yes | Yes |
| 20150629KD100.1 | Pass | Pass | 92.3 | Yes | Yes | Yes | Yes |
| 20150629KD101.1 | Pass | Pass | 94.9 | Yes | Yes | Yes | Yes |
| 20150629KD96.2 | Pass | Pass | 85.6 | Yes | Yes | Yes | Yes |

*CLP-FTIR signals; Amide A: ≈3300 cm^-1^; Amide B: ≈3080 cm^-1^; Amide I: ≈1660 cm^-1^; Amide II: ≈1500 cm^1^ [10]

Notes: Optical transmission properties greater than or equal to the transmission of the human cornea are considered acceptable. All implants met the acceptance criteria of normalized %T ≥ 95% for 500-999 nm light. Flexure tests, performed manually with pressing the implant with tweezers until ½ cm between the forks, exhibited no identifiable differences among all the implants until 12 months compared to freshly prepared implant. At 12 months the size of the implant remained constant at 14 mm diameter and FTIR showed that samples maintained the essential amide bonds from CLP inferring maintenance of both structural and chemical integrity of the implants.

**
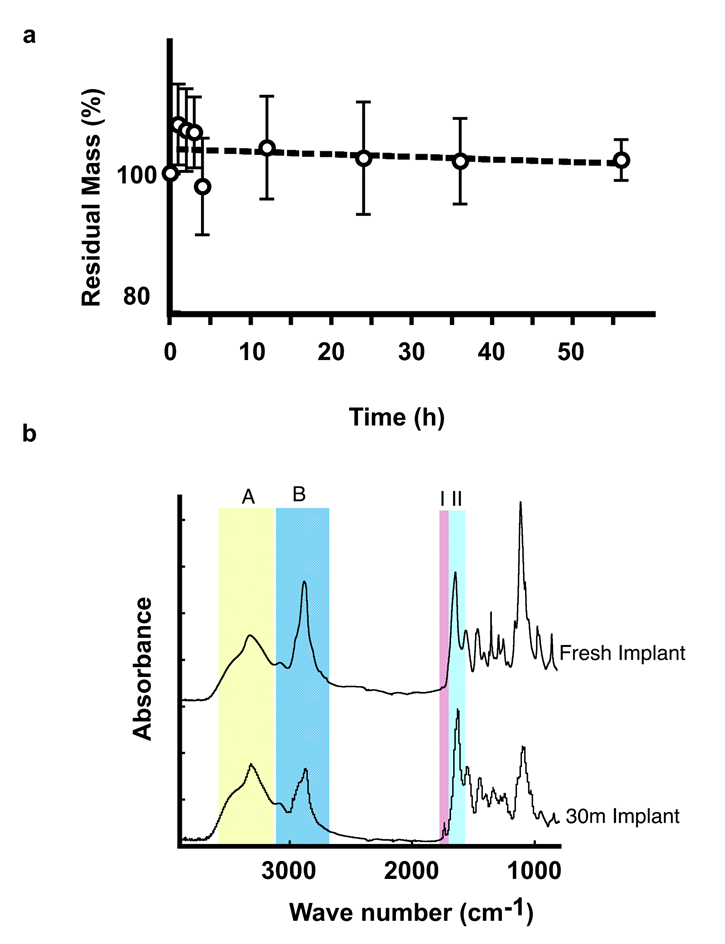
Supplementary Figures**

**Fig. S1.** Stability of CLP-PEG hydrogels over time. **(a)** Collagenase digestion profile of CLP-PEG hydrogels after 30 months of storage, showing minimal degradation when exposed to 5U/ml type I collagenase. **(b)** Representative FTIR spectrum for freshly prepared and 30-month old CLP-PEG implants. The different regions of the amide vibration from CLP used for determining the presence of the peptide within the implant, Amides A, B, I and II, are highlighted. These absorption peaks are present in both spectra. Note that FTIR is a qualitative technique, i.e. peaks are present or absent.

**Fig. S2.** Corneal thickness changes in mini-pig eyes grafted with CLP-PEG and RHCIII-MPC implants compared to contralateral unoperated eyes over the 12-month follow-up period. Measurements were made using a pachymeter. * denotes statistical significance (p≤0.05) compared to unoperated eyes by the Kruskal-Wallis test followed by the Bonferroni post-hoc test.

**
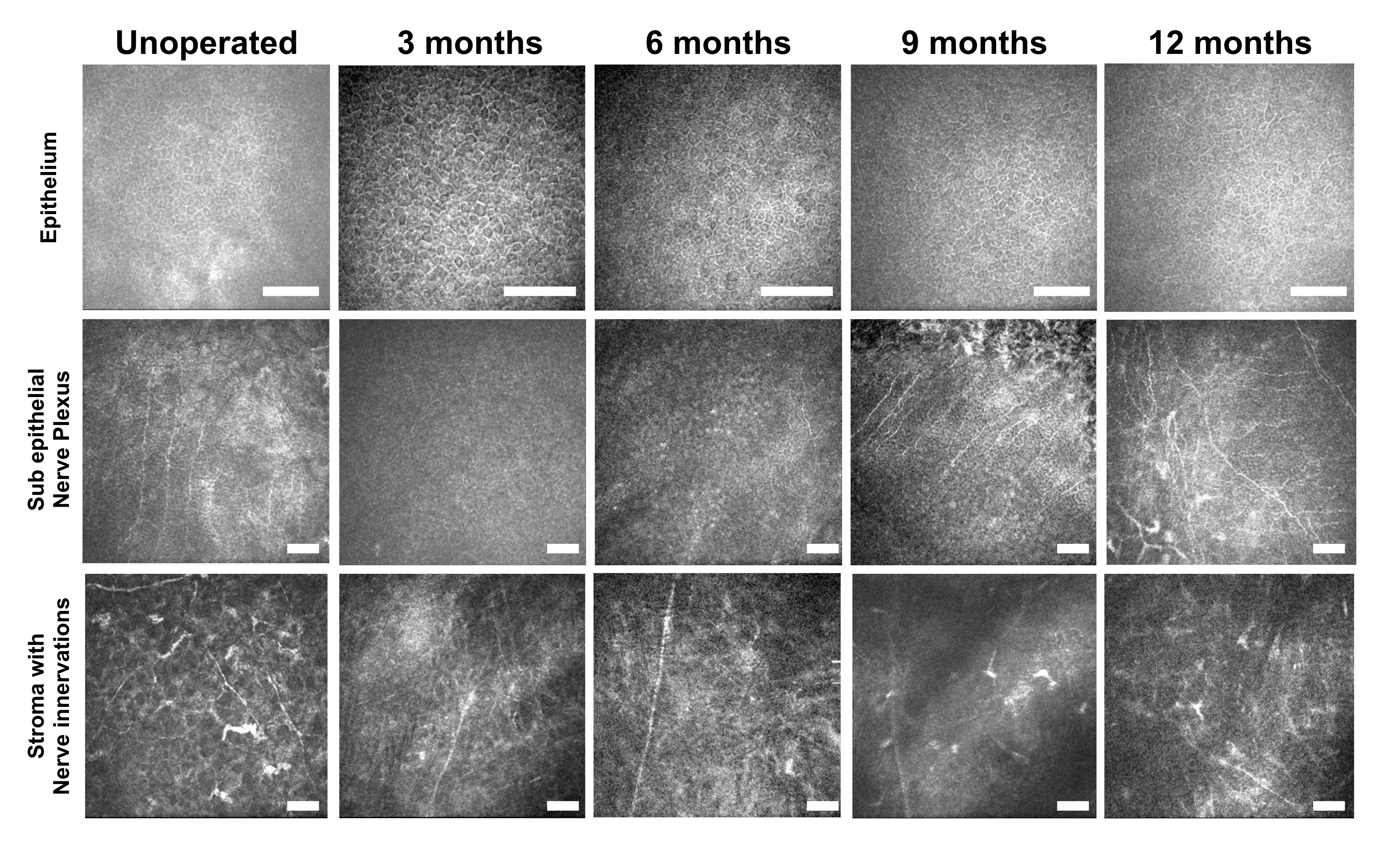
**

**Fig. S3.** IVCM images of mini-pig corneas at 3, 6, 9 and 12 months after the surgery: 1^st^ row – regenerated epithelium seen by 3 months after the surgery, which was morphologically similar to that of the control unoperated cornea (right column); 2^nd^ row – level of sub-basal nerve plexus (depth 60-80 um). Nerves were not present at 3 months after the surgery. They started penetrating the implant area by 6 months post-operation (arrow) and progressively increased in both length and density over time; 3^rd^ row – level of anterior stroma. Stromal cells had started to migrate into the implants by 3 months post-operation. Nerves seen are likely remnants of nerves severed during the surgery.


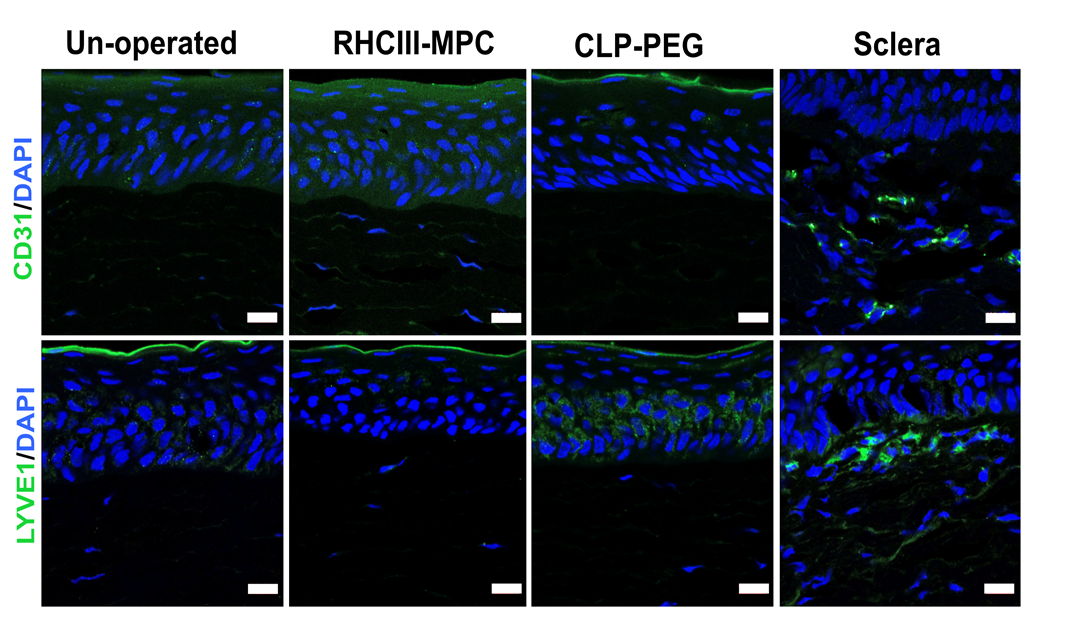


**Fig. S4.** **Immunohistochemical staining for blood and lymphatic vessels in the ocular surface.** Healthy, unoperated corneas have no blood and lymphatic vessels and hence, show no reactivity with the CD31 antibody which stains vascular endothelial cells, or the LYVEI antibody which marks lymphatic vessels. Regenerated neo-corneas in RHCIII-MPC and CLP-PEG implanted eyes were also negative for blood and lymphatic vessels. The vascularized adjacent sclera (positive control) shows positively stained blood and lymphatic vessels. Cell nuclei are stained blue with DAPI. Scale bars, 10µm.

**
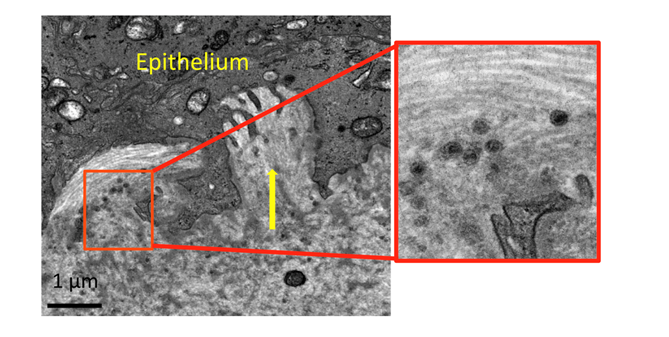
**

**Fig. S5.** TEM micrograph basal epithelial cells in regenerated CLP-PEG-grafted cornea, showing secreted extracellular vesicles. The enlargement shows membrane bound secreted vesicles located together with collagen fibrils of the corneal stromal extracellular matrix. Scale bar, 1 µm.

**
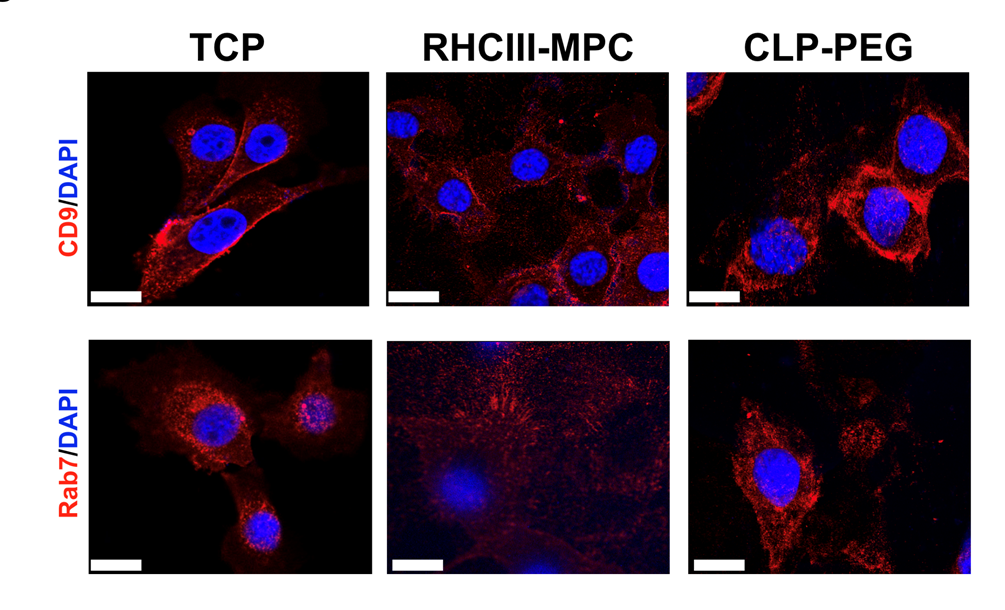
**

**Fig. S6.** Membrane bound vesicles produced by human corneal epithelial cells on tissue culture plastic (TCP), RHCIII-MPC and CLP-PEG hydrogels. Differential expression of exosome and endosome markers CD9 and Rab7 respectively are seen on the different substrate. CD9 expression is much higher in cells grown on CLP-PEG than RHCIII-MPC, while cells on TCP show expression at the leading edge of migrating cells and the periphery. Rab7 expression is cytoplasmic with stronger staining shown by cells on TCP and CLP-PEG. Nuclei are counterstained blue with DAPI. Scale bars, 25µm.

**Supplementary Bibliography**

[1] O. Buznyk, N. Pasyechnikova, M.M. Islam, S. Iakymenko, P. Fagerholm, M. Griffith, Bioengineered Corneas Grafted as Alternatives to Human Donor Corneas in Three High-Risk Patients, Clin Transl Sci 8(5) (2015) 558-62.

[2] M. Mirazul Islam, V. Cepla, C. He, J. Edin, T. Rakickas, K. Kobuch, Z. Ruzele, W.B. Jackson, M. Rafat, C.P. Lohmann, R. Valiokas, M. Griffith, Functional fabrication of recombinant human collagen-phosphorylcholine hydrogels for regenerative medicine applications, Acta Biomater 12 (2015) 70-80.

[3] M.M. Islam, R. Ravichandran, D. Olsen, M.K. Ljunggren, P. Fagerholm, C.J. Lee, M. Griffith, J. Phopase, Self-assembled collagen-like-peptide implants as alternatives to human donor corneal transplantation, RSC Advances 6(61) (2016) 55745-55749.

[4] J. Doutch, A.J. Quantock, V.A. Smith, K.M. Meek, Light transmission in the human cornea as a function of position across the ocular surface: theoretical and experimental aspects, Biophysical journal 95(11) (2008) 5092-9.

[5] T.J. van den Berg, K.E. Tan, Light transmittance of the human cornea from 320 to 700 nm for different ages, Vision Res 34(11) (1994) 1453-6.

[6] S. Patel, J. Marshall, F.W. Fitzke, 3rd, Refractive index of the human corneal epithelium and stroma, Journal of refractive surgery (Thorofare, N.J. : 1995) 11(2) (1995) 100-5.

[7] K. Merrett, P. Fagerholm, C.R. McLaughlin, S. Dravida, N. Lagali, N. Shinozaki, M.A. Watsky, R. Munger, Y. Kato, F. Li, C.J. Marmo, M. Griffith, Tissue-engineered recombinant human collagen-based corneal substitutes for implantation: performance of type I versus type III collagen, Invest Ophthalmol Vis Sci 49(9) (2008) 3887-94.

[8] H. Davson (Ed.), The Eye, Academic Press, New York (1962), p. 296.

[9] R.A. Crabb, E.P. Chau, M.C. Evans, V.H. Barocas, A. Hubel, Biomechanical and microstructural characteristics of a collagen film-based corneal stroma equivalent, Tissue Eng 12(6) (2006) 1565-75.

[10] B.B. Doyle, E.G. Bendit, E.R. Blout, Infrared spectroscopy of collagen and collagen-like polypeptides, Biopolymers 14(5) (1975) 937-57.
